# Dietary iron restriction leads to a reduction in hepatic fibrosis in a rat model of non-alcoholic steatohepatitis

**DOI:** 10.1242/bio.040519

**Published:** 2019-05-15

**Authors:** Naomichi Abe, Takuma Tsuchida, Shin-Ichiro Yasuda, Kozo Oka

**Affiliations:** Sohyaku. Innovative Research Division, Mitsubishi Tanabe Pharma Corporation, 2-2-50, Kawagishi, Toda-shi, Saitama 335-8505, Japan

**Keywords:** CDAA, Fibrosis, Iron overload, Iron-restricted diet, NASH

## Abstract

Iron overload in the liver causes oxidative stress and inflammation, which result in organ dysfunction, making it a risk factor for non-alcoholic steatohepatitis (NASH) and hepatocellular carcinoma. We aimed to evaluate the effect of dietary iron restriction on disease progression in rats fed a choline-deficient L-amino acid-defined (CDAA) diet. Male F344 rats were fed a choline-sufficient amino acid-defined (control) diet, a CDAA diet or an iron-restricted CDAA diet for 4, 8 and 12 weeks. At each time point, hepatic iron levels, oxidative stress, inflammation and fibrosis were evaluated by immunohistochemistry. The iron-restricted CDAA diet significantly decreased serum iron levels for 12 weeks compared with the CDAA diet. Histological analysis confirmed that feeding with the CDAA diet induced hepatic iron overload and that this was associated with oxidative stress (number of 8-hydroxydeoxyguanosine-positive cells), inflammation (CD68 positive area) and fibrosis (Sirius Red positive area). Iron restriction with the CDAA diet significantly led to a reduction in the hepatic iron levels, oxidative stress, inflammation and fibrosis. Therefore, dietary iron restriction could be a useful therapeutic approach for NASH patients with hepatic iron overload.

## Abstract (precis)

**Summary:** We reveal that dietary iron restriction leads to a reduction in hepatic inflammation, oxidative stress and fibrosis in rats fed a choline-deficient L-amino acid-defined (CDAA) diet.

## INTRODUCTION

Iron facilitates the formation of reactive oxygen species (ROS), and these increase cytotoxicity through lipid peroxidation, protein denaturation and DNA damage (Bacon and Britton, 1990; Olynyk et al., 2005; Pietrangelo, 2016). Excess iron in tissues therefore causes oxidative stress and inflammation, leading to organ dysfunction. For example, in patients with hereditary hemochromatosis, where iron overload is caused by a deficiency of the *Hfe* gene, iron absorption in the duodenum is increased, resulting in excessive iron accumulation in the liver and ultimately tissue damage and fibrosis (Andrews, 1999). In addition, iron overload is associated with an increased risk of hepatocellular carcinoma (HCC) (Kowdley, 2004; Pietrangelo, 2009). Iron reduction therapies, such as phlebotomy and iron chelation, are widely used for patients with disorders of iron overload (Bacon et al., 2011; European Association for the Study of the Liver, 2010; Musallam et al., 2013).

Iron overload has been reported in 30%–70% of patients with non-alcoholic fatty liver disease (NAFLD) and non-alcoholic steatohepatitis (NASH) (Fargion et al., 2001; George et al., 1998; Sumida et al., 2003). Ferritin is an iron-storage protein that is induced in response to iron overload. Serum ferritin levels increase when induced and they have been reported to predict histologic severity and advanced fibrosis in NAFLD (Kowdley et al., 2012). Iron appears to play an important role in disease progression in patients with NASH, where increased iron absorption from the duodenum is a driving factor in iron accumulation in the liver (Hoki et al., 2015). Indeed, in patients with NASH and iron overload, therapeutic phlebotomy has been shown to improve the serum alanine transaminase (ALT) level and NAFLD activity score. Iron depletion by phlebotomy has also been shown to improve insulin resistance in patients with NAFLD and hyperferritinemia (Valenti et al., 2007). These reports suggest that any form of iron reduction therapy could be a promising approach in the treatment in NASH.

Preclinical models have reported that iron chelators, such as deferoxamine (DFO) and deferasirox (DFX), have a preventive effect on disease progression induced by a choline-deficient L-amino acid-defined (CDAA) diet in rats (Kaji et al., 2011; Sakaida et al., 1999). DFO treatment significantly reduced hepatic iron levels and ameliorated oxidative stress, effectively decreasing the number of preneoplastic lesions in the livers in CDAA-fed rats. DFX significantly decreased oxidative stress, neovascularization and the activation of hepatic stellate cells in rats fed a CDAA diet. In addition, dietary iron restriction during a CDAA diet was shown to mitigate the extent of hepatic oxidative stress in rats, including DNA and lipid peroxidation (Yoshiji et al., 1992). However, the effect of simple dietary iron restriction on the progression of hepatic fibrosis and other histological features has yet to be clarified in NASH.

In this study, we aimed to evaluate the therapeutic effect of dietary iron restriction on hepatic iron, oxidative stress, inflammation and fibrosis in a rat model of NASH induced by a CDAA diet.

## RESULTS

### Dietary iron restriction was associated with a decrease in the serum iron and ALT levels

**Fig. 1.**
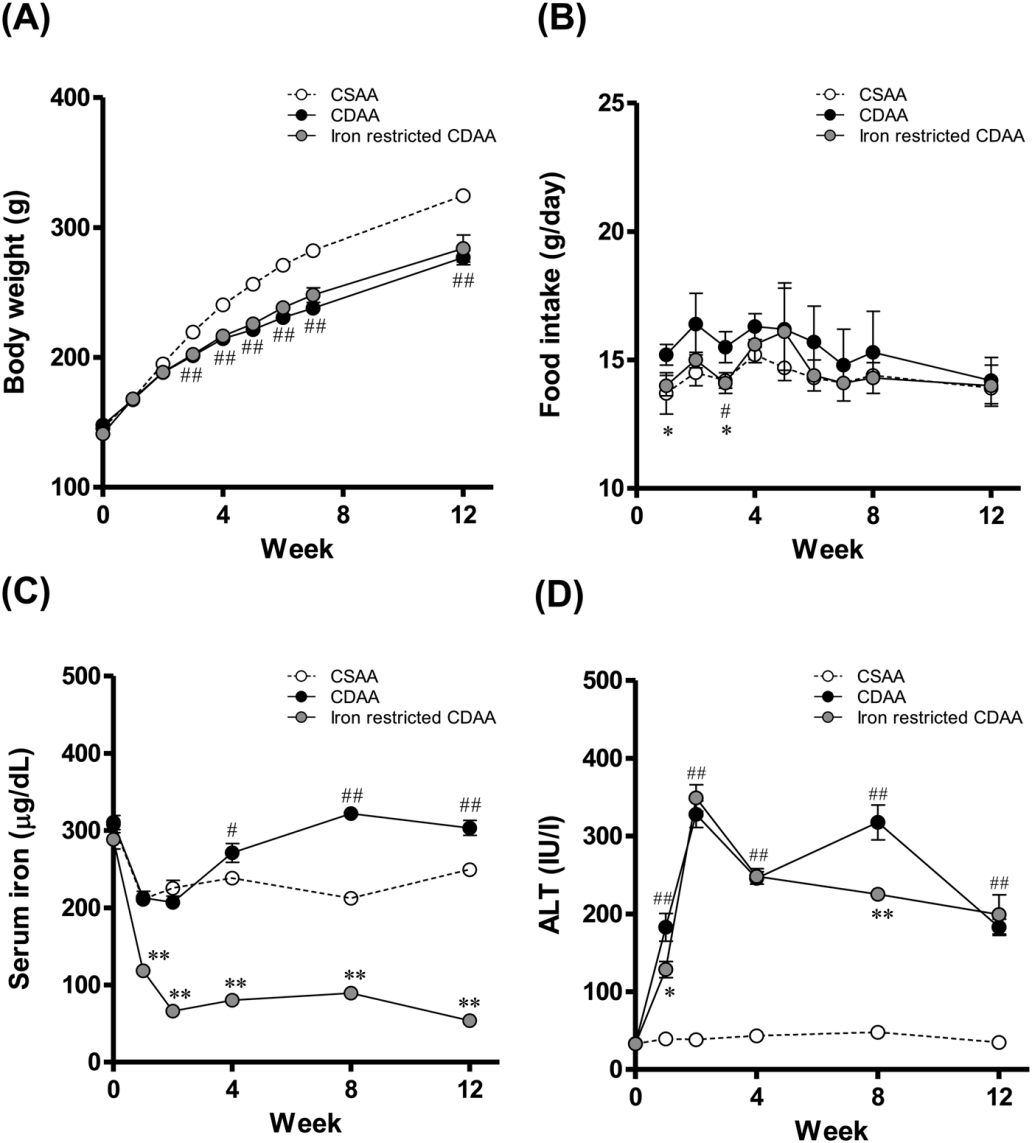
**Body weight, food intake, serum iron levels and serum ALT changes at 12 weeks in rats fed CSAA, CDAA and iron-restricted CDAA diets.** (A) Body weight. (B) Food intake. (C) Serum iron levels. (D) Serum ALT levels. Values are mean±s.e.m., *n*=18 (0–4 weeks), *n*=12 (5–8 weeks) and *n*=6 (9–12 weeks). ^#^*P*<0.05, ^##^*P*<0.01 CDAA diet-group versus CSAA diet-group. *P<0.05, **P<0.01 CDAA group versus iron-restricted CDAA group (Student's *t*-test).

In this study, only male rats were evaluated. The body weights of rats fed a CDAA diet were significantly lower than those fed a choline-sufficient amino acid-defined (CSAA) diet during the dietary interventions. Iron restriction did not affect body weight in rats fed a CDAA diet (Fig. 1A). There was no significant difference in food intake among the three dietary groups (CSAA, CDAA and iron-restricted CDAA diets; Fig. 1B). Serum iron levels in the CDAA-fed rats were significantly higher than in the CSAA-fed rats at 4, 8 and 12 weeks. The elevated serum iron induced by a CDAA diet suggests that choline deficiency could affect iron balance in the body because the same amounts of iron were present in the CDAA and CSAA diets. The iron-restricted CDAA diet produced a significant decrease in serum iron levels compared with the CDAA diet (Fig. 1C). Serum ALT levels were significantly higher in the CDAA-fed rats compared with the CSAA-fed rats, but the iron-restricted CDAA diet produced a significant decrease in serum ALT levels compared with the CDAA diet (Fig. 1D).

### Dietary iron restriction was associated with a decrease in the hepatic iron and serum ferritin levels

**Fig. 2.**
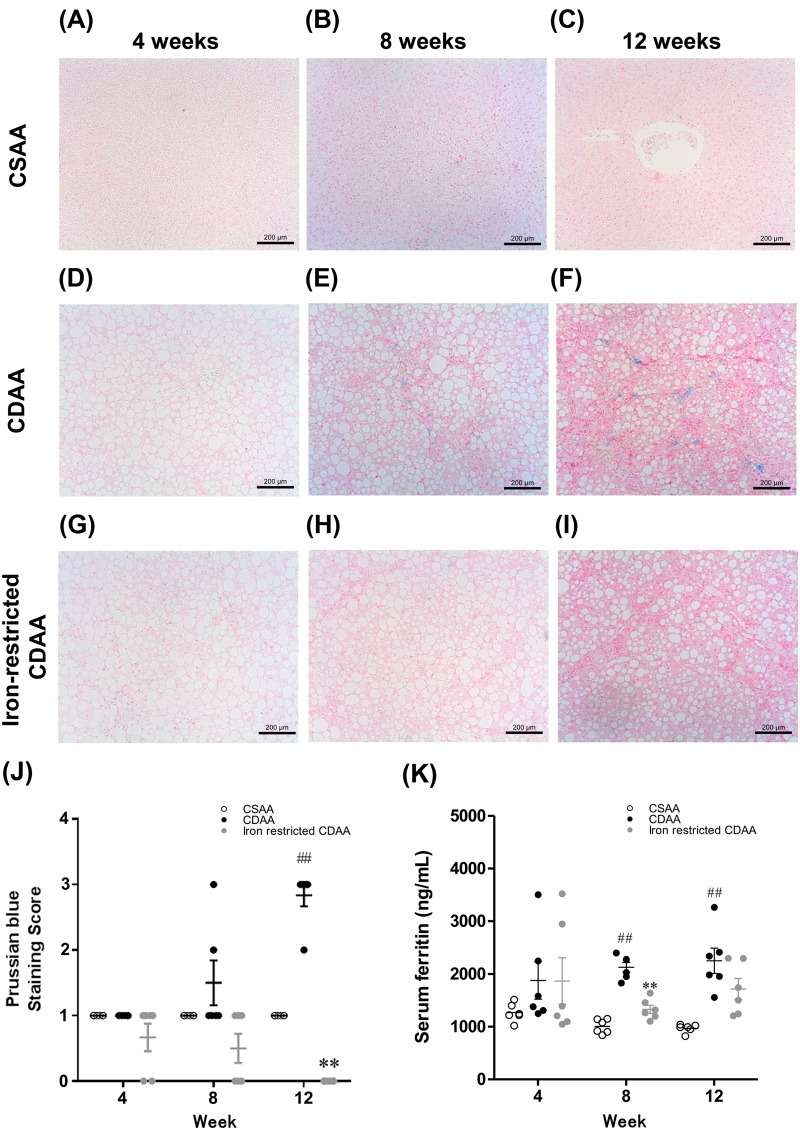
**Representative Prussian Blue-stained liver sections and staining scores at 4, 8 and 12 weeks in rats fed CSAA, CDAA and iron-restricted CDAA diets.** (A–I) Typical Prussian Blue-stained liver sections. (J) Prussian Blue staining score. (K) Serum ferritin levels. Values are mean±s.e.m., *n*=6. ^##^*P*<0.01 CDAA group versus CSAA group. **P<0.01 CDAA group versus iron-restricted CDAA group (Student's *t*-test). Scale bars: 200 μm.

Representative images of Prussian Blue-stained liver sections are shown for each group at 4, 8 and 12 weeks in Fig. 2A–I. Hepatic iron deposition and serum ferritin levels in the CDAA-fed rats were significantly increased compared with those in the CSAA-fed rats, despite that the same amounts of iron in the diets (Fig. 2J,K). Thus, dietary iron absorption from the gut was upregulated in CDAA-fed rats compared with CSAA-fed rats. In CDAA-fed rats, iron deposition was especially noted in cells of the reticuloendothelial system. Overall, our results indicate that CDAA-fed rats represented models of NASH associated with hepatic iron overload, and that dietary iron restriction significantly led to a reduction in hepatic iron deposition and serum ferritin levels in these rats (Fig. 2J,K).

### Dietary iron restriction did not affect the NAFLD activity score

**Fig. 3.**
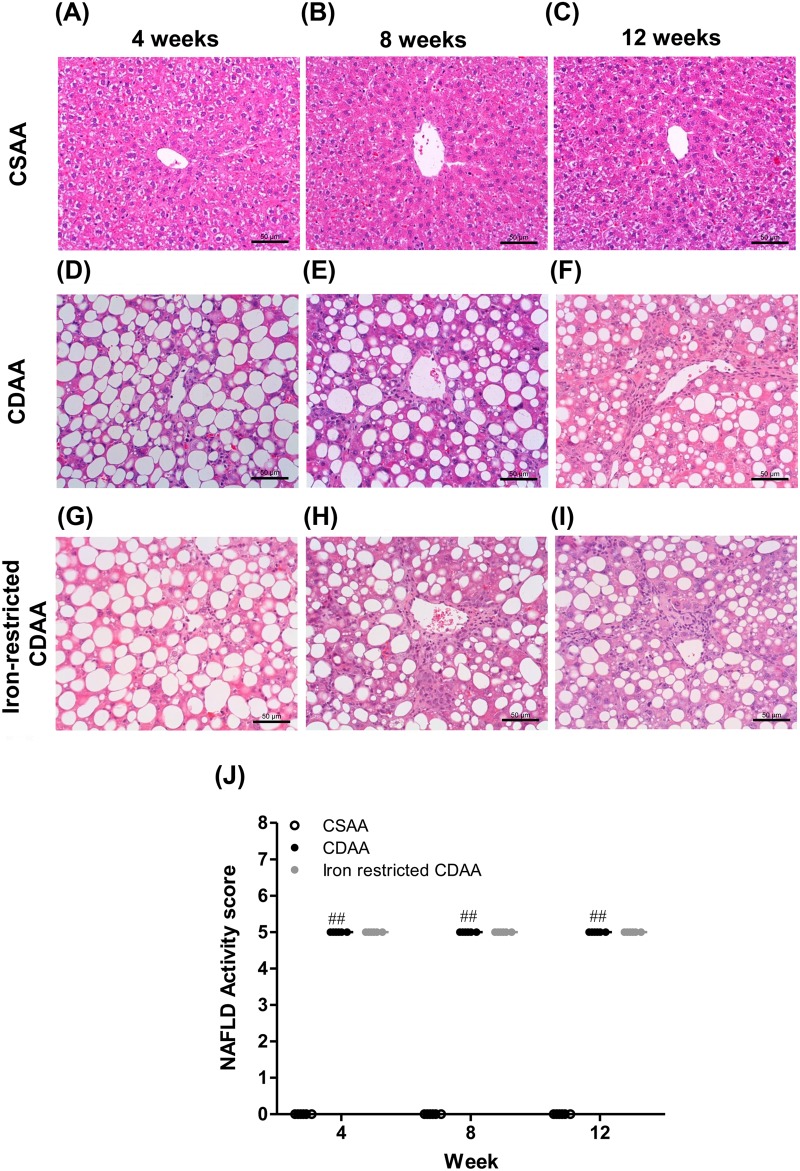
**Representative HE-stained liver sections and total NAFLD activity scores at 4, 8 and 12 weeks in rats fed CSAA, CDAA and iron-restricted CDAA diets.** (A–I) Typical images of HE-stained liver sections. (J) Total NAFLD activity score. Values are mean± s.e.m., *n*=6. ^#^^#^*P*<0.01 CDAA group versus CSAA group (Student's *t*-test). Scale bars: 50 μm.

Representative images of Hematoxylin and Eosin (HE)-stained liver sections are shown per group at 4, 8 and 12 weeks in Fig. 3A–I. The steatosis and inflammation scores in CDAA-fed rats reached a maximum of 3 points at 4 weeks and remained unchanged until 12 weeks. There were no significant differences between the CDAA and iron-restricted rats at 4, 8 or 12 weeks. No ballooned hepatocytes were observed in any group (Fig. 3J).

### Dietary iron restriction was associated with a decrease in the number of hepatic 8-OHdG-positive cells

**Fig. 4.**
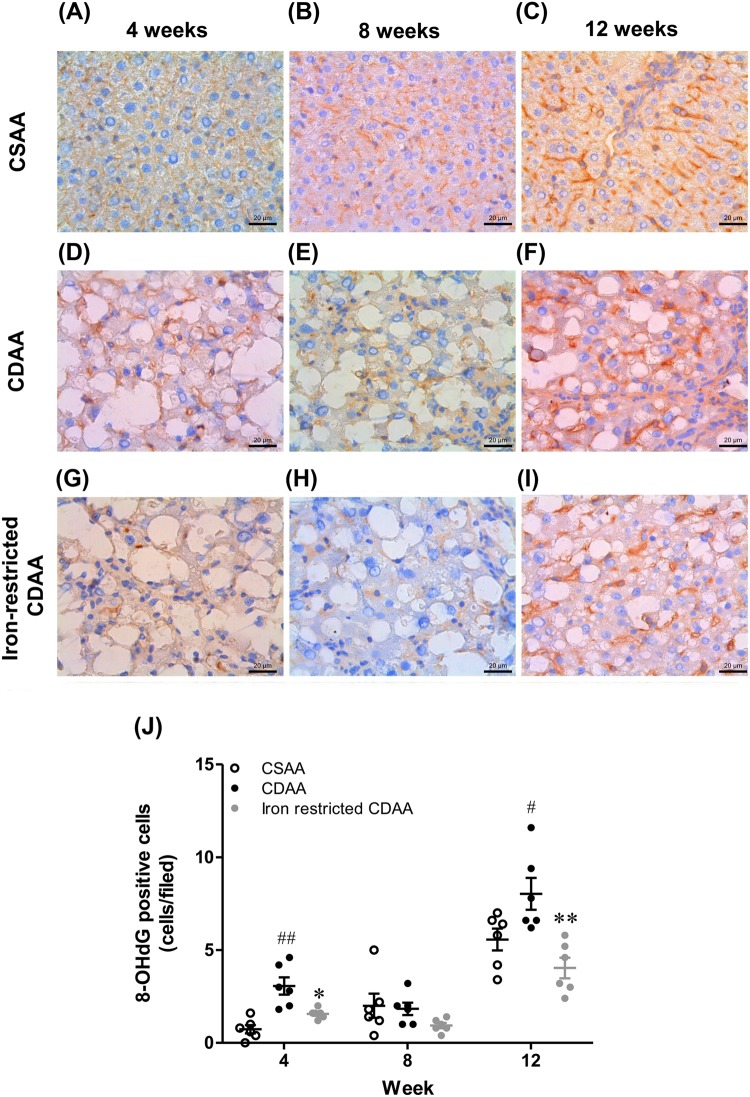
**Representative 8-OHdG-stained liver sections and positive cells at 4, 8 and 12 weeks in rats fed CSAA, CDAA and iron-restricted CDAA diets.** (A–I) Typical images of 8-OHdG stained liver sections. (J) The number of 8-OHdG positive cells. Values are mean±s.e.m., *n*=6. ^#^*P*<0.05, ^##^*P*<0.01 CDAA group versus CSAA group. **P<0.01 CDAA group versus iron-restricted CDAA group (Student's *t*-test). Scale bars: 20 μm.

Representative images of 8-hydroxydeoxyguanosine (8-OHdG) stained liver sections are shown per group at 4, 8 and 12 weeks in Fig. 4A–I. The number of hepatic 8-OHdG positive cells in CDAA-fed rats was significantly increased in comparison with those in CSAA-fed rats. The iron-restricted CDAA diet produced a significant decrease in the number of hepatic 8-OHdG positive cells compared with the CDAA diet (Fig. 4J). Thus, dietary iron restriction was associated with a decrease in oxidative stress in the livers of CDAA-fed rats.

### Dietary iron restriction was associated with a decrease in the number of hepatic CD68-positive macrophages

**Fig. 5.**
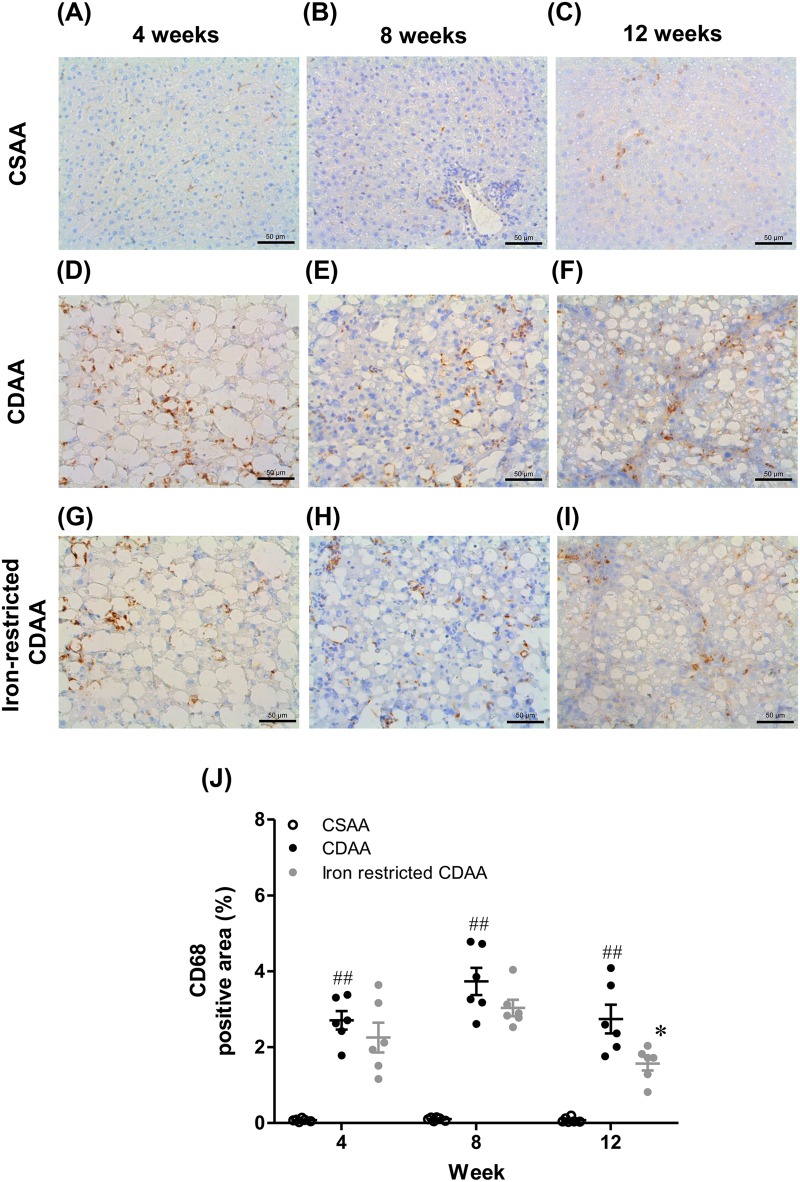
**Representative CD68-stained liver sections and positive macrophages at 4, 8 and 12 weeks in rats fed CSAA, CDAA and iron-restricted CDAA diets.** (A–I) Typical images of CD68 stained liver sections. (J) CD68 positive area. Values are mean± s.e.m., *n*=6. ^##^*P*<0.01 CDAA group versus CSAA group. *P<0.05 CDAA group versus iron-restricted CDAA group (Student's *t*-test). Scale bars: 50 μm.

Representative images of CD68 stained liver sections are shown for each group at 4, 8 and 12 weeks in Fig. 5A–I. The number of CD68-positive macrophages in the livers of CDAA-fed rats was significantly increased compared with CSAA-fed rats and the iron-restricted CDAA diet resulted in a significant decrease of that number compared with the CDAA diet (Fig. 5J). There were no differences in the inflammation score of the NAFLD activity score between the CDAA-fed and iron-restricted groups (Fig. 3). However, using the CD68 positive area, we observed quantitative inflammatory changes in macrophages by excluding other inflammatories (e.g. neutrophils), which could be counted in the NAFLD score.

### Dietary iron restriction was associated with a decrease in hepatic fibrosis

**Fig. 6.**
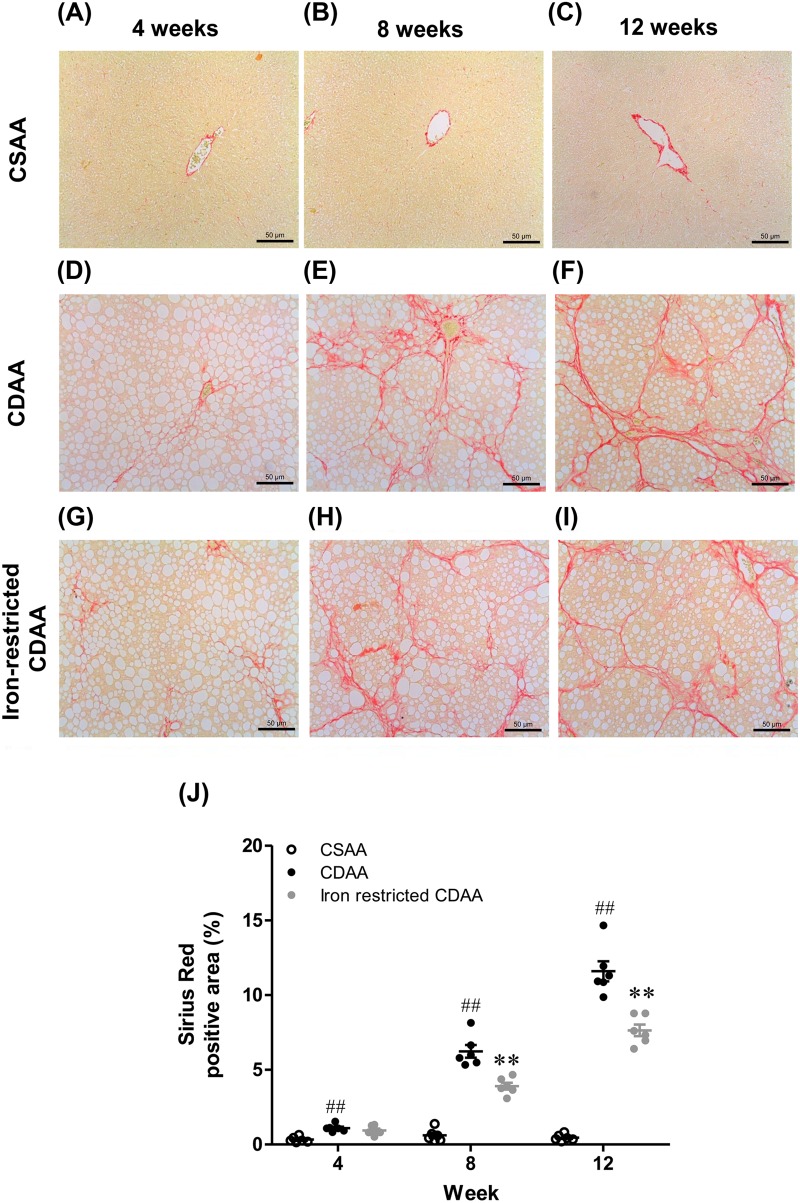
**Representative Sirius Red-stained liver sections and positive areas at 4, 8 and 12 weeks in rats fed CSAA, CDAA and iron-restricted CDAA diets.** (A–I) Typical images of Sirius Red-stained liver sections. (J) Sirius Red positive area. Values are mean± s.e.m., *n*=6. ^#^^#^*P*<0.01 CDAA group versus CSAA group. **P<0.01 CDAA group versus iron-restricted CDAA group (Student's *t*-test). Scale bars: 50 μm.

Representative images of liver sections stained with Sirius Red are shown for each group at 4, 8 and 12 weeks in Fig. 6A–I. The Sirius Red positive areas in the livers of CDAA-fed rats were significantly increased compared with the CSAA-fed rats. Bridging fibrosis was also observed in the livers of CDAA-fed rats at 12 weeks. Notably, the iron-restricted CDAA diet significantly decreased the Sirius Red positive area compared with the CDAA diet (Fig. 6J).

## DISCUSSION

We treated male F344 rats with either a CSAA diet, a CDAA diet or an iron-restricted CDAA diet for 12 weeks. Overall, our results indicated that dietary iron restriction significantly led to a reduction in hepatic iron deposition, oxidative stress, inflammation and fibrosis progression in a rat model of CDAA-induced NASH.

Hydroxyl radicals are generated by the reaction of ferrous iron, ferric iron and hydrogen peroxide. ROS, such as hydroxyl radicals and superoxide, are highly reactive to lipid, DNA, RNA and proteins, and cytotoxicity can be induced by the resulting lipid peroxidation, DNA damage and protein denaturation. Patients with iron overload disorders, such as hemochromatosis and thalassemia, suffer excessive iron deposition in the liver that results in tissue damage and fibrosis (Bacon and Britton, 1990; Pietrangelo, 2002). The reaction of hydroxyl radicals with guanosine creates 8-OHdG, which induces DNA damage and cytotoxicity, and may contribute to the development of inflammation and fibrosis in NAFLD (Cheng et al., 1992; Kitada et al., 2001). Notably, 8-OHdG levels have been shown to be increased in patients with NASH and HCC compared with those without HCC. Thus, iron-induced excess in 8-OHdG may contribute to the development of HCC in patients with NASH (Tanaka et al., 2013).

We found that feeding rats a CDAA diet significantly increased iron deposition, numbers of 8-OHdG positive cells and CD68 positive macrophages, resulting in fibrosis development. It has been reported that increased dietary iron absorption is responsible for excess hepatic iron accumulation in patients with NASH (Valenti et al., 2012). In this study, both serum iron levels and hepatic iron deposition increased significantly in the CDAA-fed rats compared with the CSAA-fed rats, despite both diets containing the same amounts of iron. Thus, we speculate that dietary iron absorption was upregulated by the CDAA diet in rats.

Hepatic iron deposition was particularly observed in reticuloendothelial cells in the CDAA-fed rats in this study. In human NASH, this has previously been associated with apoptosis and oxidative stress (Maliken et al., 2013). We speculate that increased iron absorption from the duodenum causes excess iron deposition in the hepatic reticuloendothelial system, causing both oxidative stress and inflammation to worsen. These hepatotoxic environments in the reticuloendothelial system might in turn exacerbate the progression of fibrosis in NASH.

In this study, an iron-restricted diet significantly decreased serum and hepatic iron, oxidative stress, inflammation and the progression of fibrosis in CDAA-fed rats. These results showing the therapeutic effects of dietary iron restriction are consistent with those of previous reports in models of fibrosis. For example, dietary iron restriction has been shown to improve hepatic iron, oxidative stress, inflammation and fibrosis induced by thioacetamide (TAA) in rats. Also, the levels of 8-OHdG in the liver, the expression levels of genes involved in hepatic fibrosis and the survival rate were all improved by restricting dietary iron in the TAA-induced model (Otogawa et al., 2008). Other studies have reported that iron chelators (e.g. DFO and DFX) had a preventive effect on CDAA-induced NASH progression in rats (Kaji et al., 2011; Sakaida et al., 1999). In these studies, treatment with the iron chelator decreased levels of oxidative stress (e.g. glutathione S-transferase, 8-OHdG and malondialdehyde) in the livers of CDAA-fed rats. Furthermore, hepatic fibrosis and hepatic stellate cell activation (e.g. the α-smooth muscle actin positive area) were improved by treatment with iron chelators (Kaji et al., 2011; Sakaida et al., 1999).

Iron depletion, typically by phlebotomy or chelation, is the gold standard therapy for iron overload disorders (Bacon et al., 2011; European Association for the Study of the Liver, 2010; Musallam et al., 2013). Although the therapeutic effects of iron depletion in patients with NASH are unclear, several studies have indicated that phlebotomy and dietary iron restriction might be effective for the treatment of patients with NAFLD and hyperferritinemia (Kaji et al., 2011; Sakaida et al., 1999; Sumida et al., 2009; Valenti et al., 2007). For example, Valenti et al. have reported that providing treatment with phlebotomy improved insulin resistance in patients with NAFLD who had mutations in the *HFE* gene for hereditary hemochromatosis that led to hyperferritinemia (Valenti et al., 2007). Iron depletion by phlebotomy decreased serum levels of liver enzymes, including ALT, aspartate transaminase (AST) and gamma-glutamyltransferase, in patients with NAFLD and hyperferritinemia (Valenti et al., 2014). Hoki et al. demonstrated significant decreases in the NAFLD activity score and hepatic 8-OHdG level by treatment with phlebotomy and dietary iron restriction for 48 months in patients with NASH who had increased intestinal iron absorption and iron overload (Hoki et al., 2015). Furthermore, Yamamoto et al. reported that serum ALT, AST and ferritin levels were significantly decreased by restricting dietary fat and iron for 6 months in patients with NAFLD (Yamamoto et al., 2007). Taken together, these show that iron depletion could be useful as a novel therapeutic approach for patients with NASH and hepatic iron overload.

There are several limitations in this study. Firstly, only male rats were evaluated in this study. Since there is a possibility that sex differences between male and female rats might affect iron metabolism and disease progression of NASH, it should be addressed in a future study. Secondly, mechanistic analysis between dietary iron restriction and improvement of disease progression in CDAA rats was limited. It has been reported that iron facilitates the formation of ROS and these increase cytotoxicity through lipid peroxidation, protein denaturation and DNA damage (Bacon and Britton, 1990; Olynyk et al., 2005; Pietrangelo, 2016). However, further analysis is needed for uncovering the mechanism between dietary iron restriction and amelioration of disease progression such as decrease in oxidative stress, inflammation and hepatic fibrosis in the future experiments.

In conclusion, NASH model of CDAA-fed rats showed excessive hepatic iron overload. Dietary iron restriction significantly decreased hepatic iron contents and was associated with the reduction in oxidative stress, inflammation and fibrosis in CDAA rats. Thus, dietary iron modification could be a therapeutic option for NASH patients with hepatic iron overload.

## MATERIALS AND METHODS

### Animals

Male F344 rats (3 weeks of age) were obtained from Japan SLC, Inc. (Tokyo, Japan) and housed in 12/12 h dark/light cycles with a room temperature of 22°C±3°C and a humidity of 50%±20%. All experimental protocols concerning the use of laboratory animals were reviewed and endorsed by the Institutional Animal Care and Use Committee of Mitsubishi Tanabe Pharma Corporation.

### Dietary interventions

At 6 weeks of age, the rats were divided into three groups according to their body weights and then treated with either a choline-sufficient amino acid-defined diet (CSAA diet, A06083102, Research Diet, United States), choline-deficient L-amino acid-defined diet (CDAA diet, A06083101, Research Diet, United States) or an iron-restricted CDAA diet (A06083105, Research Diet, United States). The CSAA, CDAA and iron-restricted CDAA diets contained 58.5 mg/kg, 58.5 mg/kg and 0.5 mg/kg of iron, respectively. Body weight and calorie intake were measured for 12 weeks. Serum was collected from tail veins at 0, 1, 2, 4, 8 and 12 weeks to measure ALT levels. In addition, six rats per group at 4, 8 and 12 weeks were anesthetized to collect liver and serum samples for histological and biochemical analysis. Liver samples were collected from the left lateral lobes.

### Serum and hepatic biochemical analysis

Serum ALT levels were measured by a DRI-CHEM 3500 (FUJIFILM, Tokyo, Japan). Serum ferritin levels were measured by a Rat Ferritin Quantification ELISA Kit (LSI Medience, Tokyo, Japan). Serum iron levels were measured by an Iron Assay Kit LS (Metallogenics Co., Ltd., Chiba, Japan).

### Liver histological analysis

Liver samples were fixed with 10% formalin, paraffin-embedded and sectioned (4 μm thickness) with an RM2255 Microtome (Leica Microsystems, Tokyo, Japan). Each liver section was stained with HE and Sirius Red. The NAFLD activity score was evaluated (Brunt et al., 2011) and Sirius Red positive area was quantified by ImageJ software (National Institutes of Health). Images were taken using a DFC-280 camera (Leica Microsystems). To evaluate liver iron levels, each liver section was stained with Prussian Blue (Wako, Tokyo, Japan) and the positive area was assessed by scoring on a scale from 0 to 4. The criteria per liver section were 0, <1%, 1%–5% and >5% for iron scores of 0, 1, 2, 3 and 4, respectively.

### Immunohistochemistry

Liver samples were frozen in an OCT compound (Sakura Finetek, Torrance, USA). Sections were then cut with a cryostat to thicknesses of 5 μm and dried on glass slides. To evaluate the CD68 positive area, liver sections were rinsed with 0.03% hydrogen peroxide solution and incubated in blocking solution (Block Ace Powder, DS Pharma Biomedical, Osaka, Japan) for 1 h at room temperature. To label macrophages, liver sections were incubated with mouse anti-rat CD68 antibody (1:100, MCA341R, AbD SeroTec, Inc., Oxford, UK) for 1 h at room temperature. After several rinses with phosphate-buffered saline (PBS), sections were further incubated with a peroxidase-labeled goat anti-mouse IgG antibody (1:100, P0447, Dako, Tokyo, Japan) for 30 min at room temperature and incubated with a chromogenic substrate (simple stain DAB, H1202, Nichirei Biosciences, Inc., Tokyo, Japan). Sections were mounted with Aquatex (108562, Merck, Germany), images were taken with a DFC-280 camera (Leica Microsystems) and the CD68 positive area was quantified by ImageJ software (National Institutes of Health).

To evaluate the 8-OHdG positive area, liver sections were rinsed with PBS and incubated in blocking solution (Block Ace Powder, DS Pharma Biomedical) for 10 min at room temperature. To label 8-OHdG, liver sections were incubated with mouse anti 8-OHdG antibody (1:100, MOG100P, Japan Institute for the Control of Aging, Shizuoka, Japan) for 1 h at room temperature. After several rinses with PBS, sections were further incubated with an HRP-labeled anti-mouse antibody (414171, Nichirei Biosciences), according to the manufacturer's instructions, and then incubated with a chromogenic substrate (simple stain DAB, H1202, Nichirei Biosciences). Sections were mounted with Aquatex (108562, Merck), images were taken using a DFC-280 camera and the number of 8-OHdG positive cells was quantified by ImageJ software.

### Statistical analyses

All values are presented as means±standard errors. Statistical significance was determined by Student’s *t-*test, using EXSUS ver. 8.1 (CAC Croit Corporation). *P*-values of <0.05 were considered statistically significant.
